# Trend analysis of the dosimetric impact of anatomical changes during proton therapy for maxillary sinus carcinoma

**DOI:** 10.1002/acm2.13391

**Published:** 2021-08-17

**Authors:** Yuki Narita, Takahiro Kato, Takashi Ono, Sho Oyama, Yuhei Yamazaki, Hisao Ouchi, Kimihiro Takemasa, Masao Murakami

**Affiliations:** ^1^ Department of Radiation Physics and Technology Southern TOHOKU Proton Therapy Center Koriyama Japan; ^2^ School of Health Sciences Fukushima Medical University Fukushima Japan; ^3^ Department of Radiation Oncology Faculty of Medicine Yamagata University Yamagata Japan; ^4^ Department of Radiation Oncology Southern TOHOKU Proton Therapy Center Koriyama Japan

**Keywords:** adaptive radiotherapy, anatomical change, maxillary sinus carcinoma, proton therapy

## Abstract

**Purpose:**

Anatomical changes, such as shrinkage and aeration, can affect dose distribution in proton therapy (PT) for maxillary sinus carcinoma (MSC). These changes can affect the dose to the target and organs at risk (OARs); however, when these changes occur during PT is unclear. This study aimed to investigate the dosimetric impact of anatomical changes during PT.

**Materials and Methods:**

Fifteen patients with MSC were enrolled in this study. Initial PT plans were generated based on initial computed tomography (CT) images. Several repeat CT images were obtained to confirm anatomical changes during PT. Evaluation PT plans were generated by copying initial PT plans to repeat CT images. The dose differences of the target and OARs were evaluated by comparing both the plans.

**Results:**

At 3–4 weeks after the initiation of PT, the target volume reduced by approximately 10% as compared with the initial volume. Consequently, the target volumes gradually varied until the end of treatment. The value of V_95_ (volume that received 95% of the prescription dose) in the clinical target volume of the evaluation PT plan was similar to that of the initial PT plan. However, the dose to OARs, such as the contralateral optic nerve, contralateral eyeball, brainstem, and optic chiasm, increased significantly from the middle to the later phases of the treatment course. In contrast, there was a slight dose difference in the ipsilateral optic apparatus.

**Conclusion:**

The trend analysis in this study showed that anatomical changes appeared 3–4 weeks after the start of PT, and the dose to the OARs tended to increase. Therefore, it is recommended to check the status of tumor 3–4 weeks after the start of treatment to avoid the deterioration of dose distribution due to these changes.

## 1 INTRODUCTION

Maxillary sinus carcinoma (MSC) is the most common cancer of the paranasal sinuses. The risk of metastasis is low, and squamous cell carcinoma (SCC) is the most common histological type. In addition, because it has few subjective symptoms, early detection is difficult; therefore, MSC is usually advanced when detected.^1^ In the past, the mainstream treatment for MSC was surgery. However, to preserve the function and form of the sinus, a combination of surgery, radiotherapy, and chemotherapy has recently become the standard.^2^

Reducing the radiation dose to organs at risk (OARs) for MSC is complicated because tumor is anatomically situated close to many OARs, such as optic nerves, eyeballs, lens, optic chiasm, and brainstem. A high‐dose prescription may result in severe toxicity to healthy tissues.^3^ So far, several radiation techniques have been used to treat MSC, and dosimetric comparison among three‐dimensional conformal radiotherapy, intensity‐modulated radiotherapy (IMRT), and volumetric‐modulated arc therapy (VMAT) has been reported.^4^, ^5^, ^6^ Most studies have concluded that IMRT and VMAT provide better dose coverage to the target and can spare the OARs better. In contrast, proton therapy (PT) can further reduce the radiation dose to the OARs.^7^, ^8^, ^9^, ^10^ Unlike photon beams, proton beams emit a uniform high‐radiation dose to the target and then fall steeply to zero dose.^11^ These characteristics substantially reduce dose to the surrounding healthy tissues while maximizing the dose to the tumor, giving it an inherent advantage over photon therapy.

Meanwhile, proton beams are sensitive to anatomical changes, such as body shape and tumor volume reduction.^12^ In particular, there are often changes in the target volume (shrinkage and aeration) during radiotherapy in the paranasal sinus region.^13^ These changes can extend the proton beam range, decreasing the dose coverage and increasing the dose to the OARs. In our previous study, it was shown that the range of proton beams varied due to the changes in the target volume for MSC during radiotherapy, and the dose to the OARs surrounding the target increased, especially in the optic chiasm and brainstem.^14^ Although this study showed that the dose to the OARs increased significantly 3–4 weeks after the start of treatment, these changes may occur earlier or later than this period, depending on the case. If these changes affect the quality of treatment, adaptive radiotherapy (ART), including repeat imaging and replanning, is required.^15^, ^16^, ^17^ Although a few studies have evaluated these changes in detail, when the changes in the target volume occur and how they affect the dose distribution and ART timing are unclear. Therefore, this study aimed to analyze the trend of the dosimetric impact of anatomical changes during PT for MSC.

## 2 METHODS AND MATERIALS

### 2.1 Patient selection

Between September 2012 and December 2019, fifteen patients with stage III–IV locally advanced MSC and treated with passive‐scattering PT (PSPT) at our institution were enrolled in this study. In all cases, the histological type was SCC. Table 1 shows the patients' characteristics. PT was combined with intra‐arterial infusion chemotherapy (IACT) for an average of six sessions (range, 4–8). The median total cisplatin dose for all patients was 250 mg/body (range, 150–480 mg/body). Several computed tomography (CT) images were taken during PT course to evaluate the effect of anatomical changes. This study was approved by the ethics committee of our institution.

**TABLE 1:** Patient characteristics

Characteristics	Value (range)
Sex	
Male	10
Female	5
Age (median)	65 (25–83) years
T classification	
T3	2
T4a	8
T4b	5
N classification	
N0	13
N1	1
N2	1
N3	0

### 2.2 Initial CT imaging and planning

Before the start of PT, an initial PT plan was created based on the initial CT images. Aquilion LB (Canon Medical Systems) was used as the CT scanner. CT images were obtained using a 1‐mm slice thick slice and a thermoplastic mask was used to immobilize the head and neck. In addition, magnetic resonance imaging with a 3‐mm thick slice was performed using the same immobilization method and registered to the CT images to delineate the target volume and OARs. For all patients, a physician manually contoured the target volume and OARs. The clinical target volume (CTV) was defined as the three‐dimensional expansion of the gross tumor volume (GTV) with a 3‐mm margin, encompassing the maxilla, floor, and medial aspect of the orbit, pterygomaxillary space, infratemporal fossa, ethmoid sinuses, and nasal cavity, except those around the ipsilateral optic apparatus. In PT planning, unlike photon beams, the planning target volume (PTV) concept was different; no margin was uniformly added to the entire CTV circumference. The margins placed on the CTV were mathematically calculated in PT.^18^ When expanding the margins placed on CTV, the penumbra and setup uncertainties (3 mm), range (3 mm), and Hounsfield unit uncertainties (HU) (3.5%) should be considered. In PSPT planning, the distal margin (DM), proximal margin (PM), lateral margin (LM), and compensator smear (CS) for each beam were calculated using the following equations^19^:

(1) DM=(0.035×distal CTV depth)+range uncertainty

(2) PM=(0.035×proximal CTV depth)+range uncertainty

(3) LM=setup uncertainty+penumbra

(4) $$\text{CS}=\text{square root}\;\left({\left[\text{target depth}\times 0.03\right]}^{2}+{\left[\text{setup uncertainty}\right]}^{2}\right)$$

These values were adjusted appropriately according to the positional relationship between the target and OARs. The average values of DM, PM, LM, and CS were approximately 6, 3, 10, and 4 mm, respectively. The LM was calculated assuming that the setup uncertainty and penumbra were 3 and 7 mm respectively. The brainstem, optic nerves, eyeballs, lens, and optic chiasm surrounding the target were contoured as the OARs. For each patient, the initial PT plans were created based on the initial CT images using a treatment planning system XiO‐M (Hitachi). A pencil beam algorithm was used for dose calculation in treatment planning. The PT plan was assumed to be irradiated by two fields of the anterior and lateral beams. The beam angle was slightly adjusted to consider the target shape and dose to the skin. The wobbler and ridge filter method, one of the passive‐scattering methods, was used to form the irradiation field. The total prescription dose was assumed to be 74 Gy relative biological effectiveness (RBE) in 37 fractions. An RBE value of 1.1 was used in this study. The initial PT plans were calculated so that 100% of the CTV received 95% of the prescription dose. The maximum dose was restricted to 110% of the prescription dose. The dose constraints to the OARs were limited to below the tolerable dose of each organ. The maximum dose to the contralateral optic nerve, contralateral eyeball, contralateral lens, optic chiasm, and brainstem was lower than 50 Gy (RBE), 45 Gy (RBE), 10 Gy (RBE), 50 Gy (RBE), and 50 Gy (RBE), respectively. These dose constraints were applied within the range where the dose coverage had not deteriorated. If the dose constraints could not be satisfied, the dose to the OARs was restricted to be as low as possible. The dose to the ipsilateral optic apparatus was kept to a minimum.

### 2.3 Repeat imaging and dosimetric comparisons

In all patients, repeat CT imaging was performed four times on an average (range, 3–7 times) to confirm the anatomical changes during PT (seven weeks in total). Sixty‐four repeat CT data sets were acquired, and the number of samples for each of the seven consecutive weeks was 9, 8, 9, 10, 11, 9, and 8, respectively. The spatial relationship of the isocenter of each CT image was established for each patient using a CT–CT image fusion based on bony anatomy and by eliminating the setup errors between the initial and repeat CT images. At this time, the initial CTV, defined on the initial CT images, was copied to the repeat CT image to evaluate the changes in the dose coverage. For evaluation, the GTV on the repeat CT images was re‐contoured to observe the changes in the target volume, and each OAR was contoured to evaluate the changes in the dose they received. The aeration volume was defined as the volume of air in the GTV. Based on the initial GTV, a relative reduction in the target volume (RRTV), including the aeration volume, was calculated using the following equation:

(5) $$\text{RRTV}=\left(1‐\frac{A}{B}\right)\times 100\;\left(\%\right)$$

here, *A* is the GTV volume in each repeat CT and *B* is the GTV volume in the initial CT.

An evaluation PT plan was created by copying and recalculating the beam configurations of the initial PT plan based on the initial CT images to each repeat CT image. The initial plan was compared to each evaluation plan to investigate the dose changes in CTV and OARs over time. The Wilcoxon matched‐pairs nonparametric test was performed to evaluate the dose differences by comparing both plans. A *p*‐value of 0.05 was considered statistically significant.

## 3 RESULTS

**FIGURE 1:**
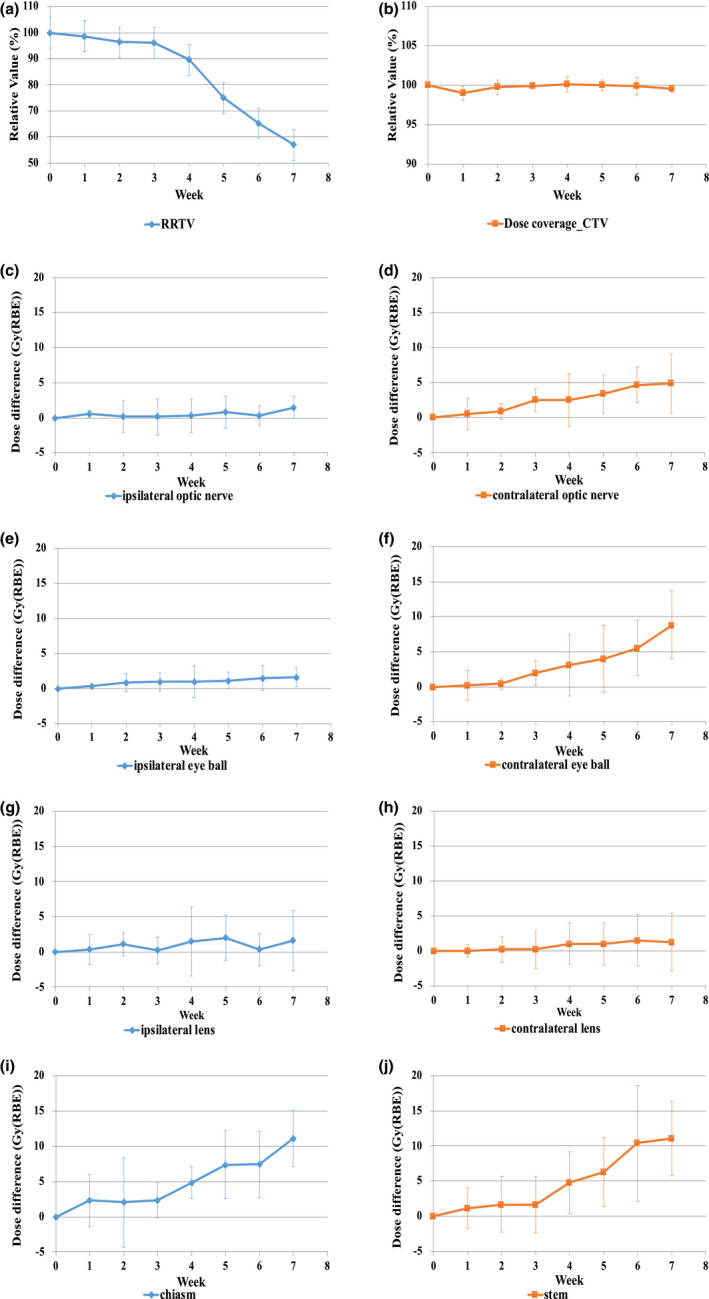
Trend of dose comparison between the initial plan and evaluation plan. (a)–(b) Weekly relative value (%) of RRTV and CTV, (c)–(j) Weekly dose difference (Gy [RBE]) in OARs. CTV, clinical target volume; RBE, relative biological effectiveness; RRTV, relative reduction of target volume

**FIGURE 2:**
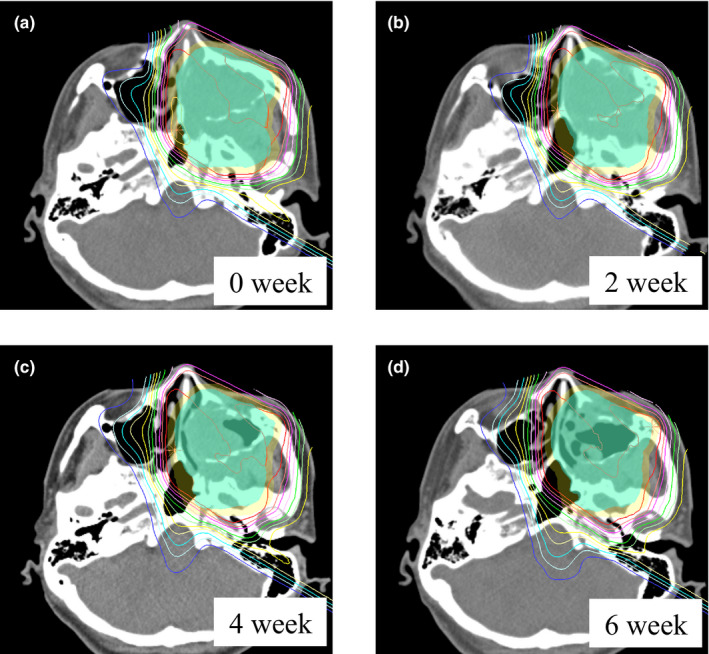
Effect of anatomical changes on dose distribution. (a) Dose distribution of the initial PT plan, (b), (c), and (d) are dose distributions at 2, 4, and 6 weeks after the start of treatment, respectively. PT, proton therapy

Changes (shrinkage and aeration) in the target volume were observed from the middle to the later phases in almost all cases. A few cases had significant changes in their body contours. Table 2 and Figure 1a show the RRTV for each week. There was a statistically significant difference in the amount of variation, and the difference was observed two weeks after the start of treatment (*p* < 0.05). Moreover, the RRTV varied by approximately 10% within 3–4 weeks of PT. There was an approximately 10%–15% variation in every week after that. Figure 2 shows the changes in dose distribution. As the treatment progressed, shrinkage and aeration were observed, and the range of the proton beam passing through this region extended greatly. Table 2 and Figure 1b show the difference in the dose to the target due to this effect. The value of V_95_ (volume that received 95% of the prescription dose) in the CTV was almost unchanged during PT, and there was no increase in the dose hot spot (the maximum dose to the CTV was <110% of the prescription dose). Consequently, the variations in the dose to the OARs were evaluated and are shown in Table 2 and Figure 1c–j. The dose to the contralateral optic nerve, contralateral eyeball, optic chiasm, and brainstem tended to increase from the middle to the later phases of the treatment course, similar to the RRTV trend. Compared with the initial PT plan, the doses to the contralateral optic nerve and eyeball in the evaluation PT plan significantly increased 3 weeks following the start of treatment (*p* = 0.027 and *p* = 0.049, respectively). The doses to the optic chiasm and brainstem significantly increased in the earlier phases of treatment (*p* = 0.042 and *p* = 0.047, respectively). These dose increases were observed continuously over the treatment course, similar to the RRTV trend. Alternatively, for the ipsilateral optic apparatus, the dose remained almost unchanged throughout the treatment course; the dose difference was not significant.

**TABLE 2:** Weekly dose comparison of the target and OARs

Factor	Week							
	0	1	2	3	4	5	6	7
RRTV	100.0 (0.0)	98.7 (1.6)	96.4 (1.9)	96.0 (2.6)	89.5 (3.5)	75.0 (13.8)	65.2 (16.2)	57.0 (20.3)
		0.143	0.021*	0.02*	0.003*	0.001*	0.001*	0.001*
Dose coverage_CTV	100.0 (0.0)	99.0 (0.7)	99.7 (0.7)	99.9 (0.1)	100.1 (0.9)	100.0 (0.6)	99.9 (0.9)	99.5 (0.4)
		0.661	0.471	0.498	0.413	0.328	0.472	0.441
Ipsilateral optic nerve	68.1 (8.5)	68.6 (3.5)	68.2 (4.1)	68.2 (3.3)	68.3 (5.0)	68.9 (6.9)	68.3 (3.2)	69.6 (3.1)
		0.374	0.423	0.477	0.65	0.111	0.233	0.307
Contralateral optic nerve	28.2 (24.7)	28.7 (13.3)	29.1 (13.3)	30.7 (9.6)	30.7 (22.4)	31.6 (19.3)	32.9 (14.5)	33.1 (15.8)
		0.460	0.306	0.027*	0.017*	0.025*	0.021*	0.035*
Ipsilateral eye ball	71.1 (8.4)	71.5 (2.4)	72.0 (3.0)	72.1 (4.6)	72.1 (4.1)	72.2 (7.2)	72.5 (4.3)	72.7 (4.7)
		0.205	0.217	0.498	0.907	0.730	0.976	0.696
Contralateral eye ball	16.9 (18.5)	17.1 (15.4)	17.3 (18.8)	18.9 (17.1)	20.0 (17.9)	20.9 (11.2)	22.5 (14.2)	25.7 (11.9)
		0.498	0.652	0.049*	0.027*	0.024*	0.001*	0.001*
Ipsilateral lens	48.8 (21.0)	49.1 (15.0)	49.9 (12.4)	49.0 (18.3)	50.2 (17.5)	50.8 (19.1)	49.1 (14.5)	50.4 (12.2)
		0.702	0.702	0.351	0.861	0.605	0.617	0.741
Contralateral lens	2.9 (5.2)	2.9 (3.0)	3.1 (3.9)	3.1 (3.4)	3.9 (4.7)	3.9 (3.6)	4.4 (4.5)	4.1 (4.7)
		0.537	0.741	0.929	0.527	0.104	0.567	0.696
Optic chiasm	37.5 (26.9)	39.8 (17.6)	39.5 (18.5)	39.8 (20.1)	42.3 (24.0)	44.8 (22.7)	44.9 (18.5)	48.6 (23.5)
		0.042*	0.035*	0.042*	0.003*	0.001*	0.001*	0.001*
Brainstem	34.0 (20.3)	35.1 (16.3)	35.6 (17.6)	35.6 (11.2)	38.7 (14.9)	40.2 (12.7)	44.3 (10.4)	45.0 (11.8)
		0.053	0.047*	0.049*	0.002*	0.001*	0.001*	0.001*

Note

The weekly dose data represent the relative value (%) of RRTV based on the initial target volume, dose coverage (%) of CTV, and maximum Gy (RBE) dose to OARs.

Each value (±standard deviation) is indicated above, and the *p*‐value is indicated below.

Abbreviations: CTV, clinical target volume; OARs, organs at risk; RBE, relative biological effectiveness; RRTV, relative reduction of target volume.

* Significant at *p* < 0.05.

## 4 DISCUSSION

In this study, we performed CT imaging several times during the PT course for MSC and observed anatomical changes over time. Also, we evaluated the dose difference of the CTV and OARs caused by these changes. The target volume changes occurred gradually after the start of treatment. In almost all cases, approximately 10% of the RRTV occurred 3–4 weeks after the start of treatment. Due to these changes, the proton beam range greatly changed when it passed through the changed region. The dose to the OARs also increased greatly (Figure 2). The dose to the OARs on the distal side of the beam increased, and the doses to the contralateral optic apparatus, optic chiasm, and brainstem tended to increase significantly (Table 2 and Figure 1c–j). Many studies have reported that these anatomical changes affected the dose to the target and OARs.^20^, ^21^ Other studies reported a similar trend in the dose changes to the OARs. However, its effect on the dose to the target was little. In this study, all PT plans were evaluated at the V_95_ of the CTV, where no target coverage deteriorated. However, when the region infiltrated by the tumor was large, and the eyeball was pressed and protruded forward, the shape of the face changed due to the shrinkage of the target volume in some cases, and the dose distribution deteriorated slightly. However, since this effect depends on the positional relationship between the target and OARs, it is unclear whether the increased dose in each case becomes a problem clinically. Although there were some cases where the dose to the ipsilateral optic apparatus exceeded the tolerable dose, the dose to other OARs was below the tolerable dose in most cases. Studies have reported that the RBE of the proton beam at the distal end was approximately 1.6–2.0 times greater than the RBE value of 1.1.^19^, ^22^ Since the maxillary sinus is a heterogeneous region where the bone structures and air regions are mixed, the range of proton beams could be altered easily, even with a slight change in the target volume. Since these changes increased the RBE value, careful attention should be given since the dose to the OARs in the distal region of the proton beam could be greater than the original plan. In actual clinical cases, it is effective to adjust the beam arrangement so that the OARs are not located on the distal side of the proton beam if distal‐end of beam changes. In this simulation study, the PT plan consisted of two fields. This design was intended to clarify the dosimetric impact of anatomical changes on the target volume. As a countermeasure, it is considered effective to increase the number of fields to order to further disperse the dosimetric impact.

Thus, anatomical changes should frequently occur during PT for MSC; however, proton beams are susceptible to these changes. In this study, we observed these changes throughout the treatment course. We showed the relationship between the expected changes in the target volume and the increased dose to the OARs. From the middle to the later phases of the treatment, tumor volume tended to change and the dose to the OARs tended to increase. Although the dosimetric impact on the target was little, the PT plan should be reviewed from the perspective of reducing the dose to the OARs at least once. However, ART, including repeat imaging and replanning for individual patients, is time consuming and labor intensive, and the optimal timing to perform ART is unclear. Based on this study, despite the variations in timing at which the dosimetric impact appeared for each OAR, it was revealed that the target volume change occurred 3–4 weeks after the start of the treatment. Since the dose distribution is expected to deteriorate during this phase, we suggest that the timing of ART should be approximately 3–4 weeks after the start of the treatment. Moreover, since this tendency is almost the same in all cases investigated in this study, these data could be used effectively when performing ART in other institutions. Currently, at our institution, routine verification CT is performed approximately 3–4 weeks after the start of treatment to confirm the status of tumors, and replanning is performed accordingly. However, considering that the target volume will change further after this timing, even if the PT plan is modified, the target volume changes may not be robust. Therefore, replanning may necessarily be done multiple times to maintain the treatment quality. In addition, the cumulative dose increase will change depending on when the replanning is performed. An appropriate number of replanning should be considered by assessing the clinical impact of each individual patient on whether the expected dose increase can be tolerated. Repeat CT imaging may be unnecessary to confirm the status of the tumor at the early phases of treatment. However, we recommend repeating the CT imaging regularly to prevent overlooking any case of tumor response progression.

In radiotherapy for MSC, photon beam therapy is another choice besides PT. In our previous study, we compared PT and VMAT regarding the effect of anatomical changes during radiotherapy, and it showed that VMAT was more robust to these changes than PT.^14^ The changes in patients' weight, body shape, and target volume during radiotherapy can be a weak point in PT; however, PT is more useful than photon beam therapy in reducing the dose to contralateral OARs. Since the two treatment modalities have advantages and disadvantages, we should select the treatment method that is appropriate for each case. Moreover, PT faces a significant challenge in reducing the dose to ipsilateral OARs; therefore, further reduction is necessary. Recently, pencil beam scanning (PBS) delivery technology has become clinically widespread due to the development of irradiation technology.^23^, ^24^ Compared to PSPT, which has a limitation in reducing the dose to OARs in cases with complex target shapes, intensity‐modulated proton therapy (IMPT) using the PBS technology can provide an even better dose coverage and dose reduction to OARs. However, studies reported that IMPT is more sensitive to organ variation and inter‐fraction variations, such as weight loss and tumor size, than PT.^25^, ^26^ Therefore, anatomical changes should have greater effect when using the IMPT delivery technology than when using the PT evaluated in this study. We plan to introduce the PBS delivery technology to our institution in the future. When treating MSC, understanding the characteristics of the PBS delivery technology and selecting the appropriate treatment method for each case are necessary.

There were several limitations in this study. First, we only evaluated the cases of SCC. There are other histological types, such as adenoid cystic carcinoma in MSC. Moreover, the changes in the target volume were greatly related to the effect of PT and chemotherapy. At our institution, IACT was used along with PT in almost all cases, and this might have caused a large change in the target volume.^27^, ^28^ Therefore, it should be noted that the tendency of reaction may differ depending on the difference in histological type and presence or absence of chemotherapy. Second, all PT plans in this study were calculated using the pencil beam algorithm. This algorithm is known to not accurately calculate the proton dose and ranges in heterogeneous regions. In other studies, the difference in the distal side of the proton beam was a result of a simulation using the Monte Carlo dose calculation algorithm.^29^, ^30^ Although the dose evaluation of CTV and OARs lacked the accuracy of Monte Carlo dose calculation, we considered that the trend of the dose to OARs was evaluated sufficiently and provided enough information.

## 5 CONCLUSION

In this study, the trend of the dosimetric impact of anatomical changes during PT for MSC was analyzed. A trend was observed that gradual changes in the target volume occur. The tumor shrank approximately 3–4 weeks after the start of treatment. The dose to the OARs increased accordingly. To deal with the deterioration of the dose distribution due to these changes during radiotherapy, it is recommended to confirm the status of tumor and to improve the dose distribution in the latter half of the treatment course.

## 7 CONFLICT OF INTEREST

The authors declare no conflict of interest.

## AUTHOR CONTRIBUTION

Yuki Narita: Study design, Investigation, Writing‐Original Draft. Takahiro Kato: Study design, Writing‐Review & Editing. Takashi Ono: Study design, Writing‐Review & Editing. Sho Oyama: Data Collection. Yuhei Yamazaki: Data Collection. Hisao Ouchi: Data Collection. Kimihiro Takemasa: Data Collection. Masao Murakami: Writing‐Review & Editing.

## Data Availability

The data that support the findings of this study are available from the corresponding author upon reasonable request.
